# A Devastating Floating Aortic Thrombus and Ketosis-Prone Diabetes

**DOI:** 10.7759/cureus.44836

**Published:** 2023-09-07

**Authors:** Ricardo A Serrano, Lavinia Kolarczyk, Daniel J Rosenkrans

**Affiliations:** 1 Anesthesiology, University of North Carolina at Chapel Hill, Chapel Hill, USA; 2 Anesthesiology and Critical Care, University of North Carolina at Chapel Hill, Chapel Hill, USA

**Keywords:** ketosis prone diabetes, aortic diseases, systemic anticoagulation, diabetic keto acidosis, aortic thrombosis

## Abstract

This article reports a case study of a middle-aged patient diagnosed with Ketosis-Prone Diabetes (KPD) and diabetic ketoacidosis who had a mobile thrombus in the distal aortic arch with catastrophic complications from thrombus embolization. The pathogenesis of the mobile aortic thrombus is currently under investigation, with many risk factors having been found. Based on the patient's limited manifestation of atherosclerosis and the absence of any indications of thrombophilia, KPD and inflammation from uncontrolled hyperglycemia likely played a significant role in the formation of the thrombus. KPD is a subtype of diabetes characterized by the abrupt onset of severe hyperglycemia and ketoacidosis. The inflammation caused by uncontrolled hyperglycemia in KPD patients can lead to endothelial dysfunction and the activation of prothrombotic pathways. There is a lack of consensus regarding the optimal approach for managing a mobile aortic thrombus. The main strategies under consideration are conservative care, including anticoagulation alone, invasive removal of the thrombus, or endovascular intervention.

## Introduction

A mobile aortic thrombus is an uncommon clinical phenomenon. Mobile thrombi are more frequently detected in the thoracic aorta and less commonly in the aortic arch. Increased use of imaging studies has led to better detection of this source of systemic embolization. The exact etiology and pathophysiology are unclear, but risk factors can be broadly categorized into vascular endothelial injury states, abnormal coagulation function disorders, malignancy, steroids, estrogen, and trauma [1]. How to manage a floating aortic thrombus is controversial. Treatment options include anticoagulation, endovascular intervention, or surgical removal, depending on the individual's condition and response to treatment [2]. We present a case report of a floating aortic thrombus and diabetic ketoacidosis in the setting of recent onset Ketosis-Prone Diabetes (KPD) ("Flatbush diabetes").

## Case presentation

**Figure 1:**
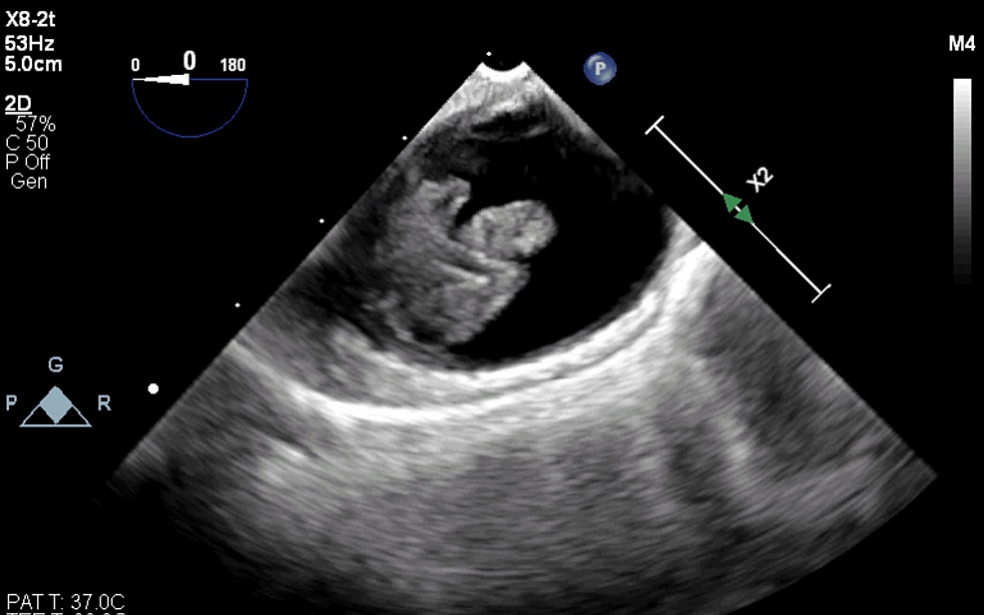
Transesophageal echocardiogram (TEE) confirms the presence of a markedly movable mass located in the distal region of the aortic arch.

A 47-year-old man of Hispanic origin with a body mass index of 28.1 kg/m^2^ presented to an outside hospital complaining of chest pain, acute onset severe abdominal pain, vomiting, and numbness of the left lower extremity. The patient's medical history was unremarkable except for dyslipidemia. Prior to admission, he was not taking any medications. He was transferred to our institution for emergent treatment of bilateral lower extremity acute limb ischemia, rhabdomyolysis, and acute renal failure. He was immediately taken to the operating room for a right popliteal and tibial artery embolectomy, right lower extremity four-compartment fasciotomies, left lower extremity iliac, femoral, and popliteal artery embolectomies and left lower extremity above the knee amputation under general anesthesia. An intraoperative transesophageal echocardiogram (TEE) showed a large, partially mobile echo density distal to the left subclavian artery. The echodensity measured approximately 2 cm x 1 cm (Figure 1).

**Figure 2:**
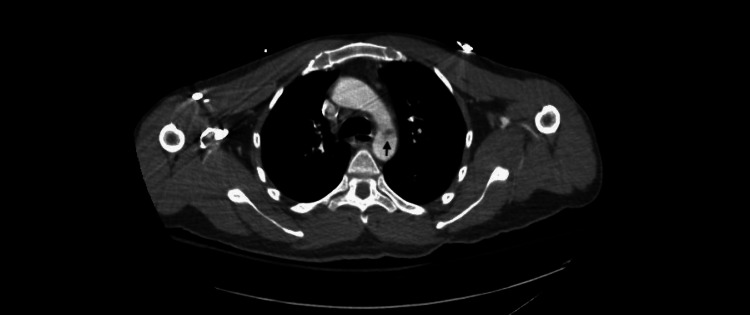
CT scan showing a filling defect in the distal aortic arch (arrow).

The diagnostic workup included a preoperative computed tomography (CT) angiography, which showed a low-density filling defect in the aortic arch at the level of the ligamentum arteriosum consistent with a non-occlusive aortic arch thrombus and multiple distal emboli occluding the left and right internal iliac arteries (Figure 2). The ascending aorta and the aortic arch had intact intima and normal size. The initial laboratory studies are summarized in Table 1. He had been started on an insulin infusion protocol to correct his diabetic ketoacidosis. He received large-volume resuscitation as initial therapy, which continued during transfer and arrival to our institution. In addition, a heparin infusion was initiated after surgery. His immediate postoperative course was complicated by significant bleeding from the amputation site, multiple blood transfusions, and worsening renal failure requiring continuous renal replacement therapy.

**Table 1:** Laboratory workup H: high; L: low; WBC: white blood cells; CK: creatine kinase

Lab	Value	Reference Range
pH, Arterial	6.93 L	7.35 – 7.45
Lactate	5.7 H	<1.3 mmol/L
HCO3	7 L	22 – 27 mmol/L
Sodium	143	135 – 145 mmol/L
Potassium	6.1 H	3.4 – 4.6 mmol/L
Anion Gap	33 H	4 – 12 mmol/L
WBC Count	23.9 H	3.6 – 11.2 x 10^9^/L
Platelet Count	405	150 – 450 x 10^9^/L
Hemoglobin	18.1 H	11.3 – 14.9 g/dL
Creatinine	1.83 H	0.60 – 0.80 mg/dL
Glucose	661 H	70 – 179 mg/dL
Beta-hydroxybutyrate	>12.6 H	0.02 – 0.27 mmol/L
CK	80,324 H	46.0 – 171.0 U/L
CK-MB	263.91 H	0.00 – 5.00 ng/ml
Troponin I	< 0.045	< 0.045 ng/mL

On postoperative day 2, he was noted to have absent Doppler signals in his right lower extremity; an arterial duplex ultrasound showed evidence of outflow obstruction and no flow to the distal posterior and anterior tibial arteries. He was taken to the operating room for repeat embolectomy of the right lower extremity. The remainder of his hospital course required completing the left above-the-knee amputation and right lower extremity wound care. The metabolic derangements continued to improve, and his insulin requirements greatly decreased.

An extensive thrombophilia workup was unremarkable, including antithrombin III levels, protein C and S, factor V Leiden mutation, prothrombin mutation, lupus anticoagulant, and antiphospholipid. In addition, the paroxysmal nocturnal hemoglobinuria assay, antineutrophilic cytoplasmic antibody (ANCA), JAK2 mutation analysis, and RPR (Rapid Plasma Reagin) for syphilis were also all negative. The patient's first episode of diabetic ketoacidosis was attributed to ketosis-prone diabetes, given his normal C-peptide levels and negative Beta-cell autoantibodies.

A subsequent magnetic resonance imaging (MRI) two weeks after presentation showed no evidence of thrombus. He continued anticoagulation with heparin and transitioned to Aspirin and Apixaban before being discharged to a rehabilitation facility.

## Discussion

The current case describes a middle-aged patient with a new diagnosis of KPD and diabetic ketoacidosis, who presented with a mobile thrombus of the distal aortic arch, manifested by aortoiliac thrombosis, needing above-the-knee amputation and revascularization. The cascade of prothrombotic events that ensued with uncontrolled hyperglycemia may have precipitated local thrombus formation in an aorta devoid of primary aortic disease, that is, without signs of aortic atherosclerosis, aneurysm, aortic dissection, or aortitis. The floating aortic thrombus was treated conservatively with systemic anticoagulation, and a full resolution was evidenced with appropriate imaging studies two weeks later.

A floating aortic arch thrombus in a nonaneurysmal, minimally atherosclerotic, or normal aorta is currently regarded as a distinct clinical entity [3]. It has a reported incidence of only 0.45% [4], and most are found in the thoracic aorta, followed by the aortic arch with a predilection for the aortic isthmus and less commonly found in the ascending aorta [5]. Mobile aortic thrombus can be sessile and/or pedunculated. A pedunculated aortic thrombus can break off more frequently with a rate of embolization of 73% as compared to 12% in sessile thrombi [6]. This obscure and underrecognized source of systemic embolization has become more readily identifiable due to the widespread use of imaging studies. The etiopathogenesis is not clear. Still, multiple risk factors have been described, including hypercoagulable states, smoking, chronic steroid or estrogen use, chronic inflammatory conditions, drug use, heparin-induced thrombocytopenia, rheumatism, primary endothelial disorders, and history of vasculitis [1]. Another potential cause for primary aortic arch thrombus may be a vulnerable plaque with subsequent local thrombosis facilitated by a hypercoagulable state [7]. The patient's history of dyslipidemia, minimal evidence of atherosclerosis, without underlying dissection or aneurysm, and negative hypercoagulability workup make his recent diagnosis of KPD a likely etiology presumably secondary to the inflammatory state and the enhanced prothrombotic condition seen in uncontrolled diabetes syndromes [8,9]. This manner of presentation has not been reported in the literature.

KPD or Flatbush diabetes is a form of diabetes that usually presents with new-onset diabetes with provoked or unprovoked diabetic ketoacidosis (DKA). The classification of KPD is determined by the presence or absence of β-cell autoantibodies (A+ or A-) and β-cell functional reserve (β+ or β-), resulting in four distinct types. KPD is prevalent in African, African American, Asian, and Hispanic populations [10]. A subset of patients with unprovoked ketosis are male and achieve excellent glycemic control after insulin discontinuation following the first episode of DKA [11]. In susceptible individuals, the triggers for the abrupt onset of hyperglycemia, coupled with or without ketoacidosis severe, remain unclear [12].

A floating aortic thrombus may be identified in individuals who exhibit symptoms following a serious embolic event. The patient's aortic thrombus was found after a computed tomography angiogram (CTA) and visualized again during the intraoperative transesophageal echocardiogram (TEE). TEE is the preferred ultrasound method for examining the thoracic aorta. It provides high-resolution images of the aortic arch, with one notable exception of a small segment of the distal ascending aorta near the innominate artery [13]. Since the entire aorta cannot be visualized with TEE, a CTA is essential for diagnosis and is a mandatory test during workup.

No guidelines or consensus are reported in the literature on managing a mobile aortic thrombus. Available case series describe success with systemic anticoagulation therapy alone, thrombolytic treatment, minimally invasive endovascular stent graft, or surgical thrombus removal with or without graft replacement [14]. Open thrombus removal with resection of the attachment site is advocated in symptomatic patients due to the high risk for recurrent embolism [15]. Stent graft exclusion could also be a viable alternative primary treatment for a symptomatic mobile thrombus of the distal aortic arch [16]. However, treating the etiology behind thrombus formation and stabilizing the thrombus with full anticoagulation may also prevent further embolization. Any interventional approach has important risks and morbidities, including embolization. For this reason, conservative management with anticoagulation can be considered a first choice, and if embolization or the thrombus persists, an endovascular or open thrombectomy can be recommended [2,17]. In our patient, a full resolution of the thrombus and no additional embolic events were documented with MRI after two weeks of full anticoagulation treatment.

## Conclusions

The clinical presentation of KPD and diabetic ketoacidosis with a mobile aortic thrombus is rare. The association of these two distinct pathologies and their causes remains unclear. CTA and TEE play essential roles in diagnosing and evaluating primary aortic diseases. Combining the information from both imaging modalities allows for a more accurate diagnosis and treatment plan. Conservative management with anticoagulation is an acceptable initial approach. This allows for treating the underlying cause and stabilizing the thrombus, reducing the risks and morbidities associated with interventional procedures. However, no guidelines or consensus exist on managing mobile aortic thrombus. Further research is needed to fully understand these two conditions' complex pathogenesis and identify effective preventive measures.
